# Proteomic profiling of serum samples from chikungunya-infected patients provides insights into host response

**DOI:** 10.1186/1559-0275-10-14

**Published:** 2013-10-14

**Authors:** Vinuth N Puttamallesh, Sreelakshmi K Sreenivasamurthy, Pradeep Kumar Singh, H C Harsha, Anjali Ganjiwale, Shobha Broor, Akhilesh Pandey, Jayasuryan Narayana, T S Keshava Prasad

**Affiliations:** 1 Institute of Bioinformatics, International Technology Park, Bangalore 560 066, India; 2 Department of Microbiology, All India Institute of Medical Sciences, New Delhi 110 029, India; 3 Microtest Innovations Pvt. Limited, International Technology Park, Bangalore 560 066, India; 4 McKusick-Nathans Institute of Genetic Medicine and Departments of Biological Chemistry, Pathology and Oncology, Johns Hopkins University School of Medicine, Baltimore 21205 MD, USA; 5 Department of Biological Chemistry, Johns Hopkins University School of Medicine, Baltimore 21205 MD, USA; 6 Department of Pathology, Johns Hopkins University School of Medicine, Baltimore 21205 MD, USA; 7 Department of Oncology, Johns Hopkins University School of Medicine, Baltimore 21205 MD, USA

**Keywords:** iTRAQ, Quantitative proteomics, Arthritis, Hyponatremia, Neuropathology

## Abstract

**Background:**

Chikungunya is a highly debilitating febrile illness caused by Chikungunya virus, a single-stranded RNA virus, which is transmitted by *Aedes aegypti* or *Aedes albopictus* mosquito species. The pathogenesis and host responses in individuals infected with the chikungunya virus are not well understood at the molecular level. We carried out proteomic profiling of serum samples from chikungunya patients in order to identify molecules associated with the host response to infection by this virus.

**Results:**

Proteomic profiling of serum obtained from the infected individuals resulted in identification of 569 proteins. Of these, 63 proteins were found to be differentially expressed (≥ 2-fold) in patient as compared to control sera. These differentially expressed proteins were involved in various processes such as lipid metabolism, immune response, transport, signal transduction and apoptosis.

**Conclusions:**

This is the first report providing a global proteomic profile of serum samples from individuals infected with the chikungunya virus. Our data provide an insight into the proteins that are involved as host response factors during an infection. These proteins include clusterin, apolipoproteins and S100A family of proteins.

## Background

Chikungunya is an endemic disease in Africa and South-East Asia. Some isolated cases have also been reported in parts of Europe, Australia and America in the past decade [1-3]. Although the infection was considered to be arthritogenic, cases of meningoencephalitis and subsequent mortality have also been documented [4-6]. The persistent and painful arthralgia observed in this disease significantly reduces the ability to work among the affected.

Chikungunya virus (CHIKV) is an arbovirus belonging to the family *Togaviridae* and genus *Alphavirus*. Its genome consists of 12 kb single-stranded RNA, which codes for 4 non-structural and 5 structural proteins. A mutation in the viral genome leading to a single amino acid change of alanine to valine at the position 226 (A226V) of the E1 glycoprotein [7], enhances its survival and transmission through the widely distributed vector- *Aedes albopictus*[8,9]. This has also been shown to enhance the dissemination of the virus into temperate regions, in addition to its tropical hot-spots in the native regions of Africa and South-East Asia [10-12].

Following the bite of an infected mosquito, the virus replicates in the skin before being disseminated to other parts of the body including liver, spleen, muscle, joints and occasionally choroid plexus of the central nervous system [4,13,14]. Clinically, the disease presents with an acute onstage of fever, rigors, headache and occasional rashes followed by severe joint pain lasting weeks to months [15,16]. It is often confused with dengue fever during the acute stage [17]. Confirmatory diagnosis of chikungunya involves the use of RT-PCR based detection of chikungunya viral transcript and/or IgG or IgM capture ELISA [18,19]. Initial screening through monitoring of interleukin levels and subsequent confirmation of viral glycoproteins by ELISA cannot be easily implemented in remote rural areas.

Various research groups have investigated the pathogenicity and the mechanism of CHIKV infection [14,20,21]. Symptoms associated with chikungunya have been proposed to be of an immunopathogenic origin caused due to cytokines. Levels of cytokines have been studied to understand the clinical manifestations observed in patients. Among them, cytokines such as IL-1 and IL-6 have been shown to be elevated in the acute stage, with persistently higher levels of IL-6 in patients with continued arthralgia [22-25]. The cytokine levels in chikungunya patients have also been associated with the severity of infection [22]. Cytokine profiles have been shown to vary with the viral loads and the stages of infection [23]. Recent advances in the development of macaque models and arthritic mouse models for chikungunya may provide an opportunity for further understanding of the disease at a molecular level [26,27]. A recent analysis of the genetic predisposition of individuals from affected families suggests that people with O + ve blood group are more susceptible to chikungunya infection. This study also found that Rh negative individuals were more resistant to chikungunya infection [28]. Although studies focusing on a limited panel of cytokines have been studied in the context of chikungunya, a more comprehensive study to investigate the entire serum proteome profile has not yet been reported. Thus, we performed a global quantitative proteomic profiling of serum samples from chikungunya patients along with unaffected controls using high-resolution mass spectrometry.

## Results and discussion

We carried out an iTRAQ-based quantitative proteomic profiling of serum samples from chikungunya patients and control individuals after immunoaffinity-based depletion of abundant serum proteins, *in vitro* labeling with iTRAQ reagents and SCX fractionation followed by high resolution Fourier transform mass spectrometry (LTQ-Orbitrap Velos). From this, we acquired a total of 112,419 tandem mass spectra, which were searched using two search algorithms - SEQUEST and Mascot - against a total of 33,987 proteins as provided in the Human RefSeq database version 52. We obtained 27,809 peptide-spectrum matches (PSMs) corresponding to 3,803 peptides and 569 proteins. Of these 569 proteins, identification of 367 proteins was supported by 2 or more peptides, while 202 protein identifications were based on single peptide evidence. A comparison of the proteomic data obtained from this study with the proteins annotated in the Plasma Proteome Database [29] confirmed that 522 of 569 proteins identified in this study have been previously reported from the serum or plasma [29]. We submitted the results of this analysis to public repositories including PRIDE (http://www.ebi.ac.uk/pride) [30] and Human Proteinpedia (http://www.humanproteinpedia.org) [31].

**Figure 1:**
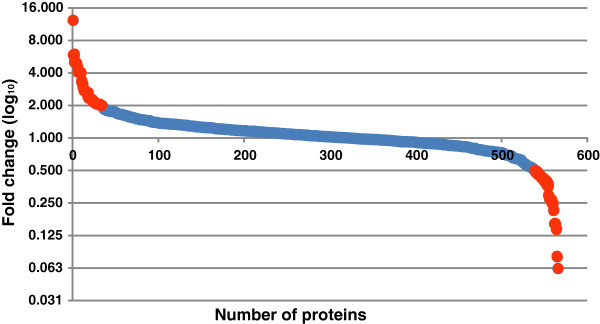
**Protein distribution.** Proteins identified are plotted against the logarithmic values of their fold change in chikungunya serum samples when compared to that of controls. Red colored dots represent the differentially expressed proteins (≥ 2-fold).

**Figure 2:**
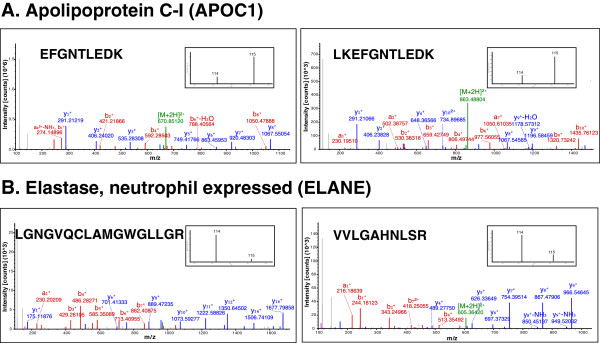
**Representative MS/MS spectra of peptides, which were differentially expressed in chikungunya serum samples as compared to control. (A)** MS/MS spectra of 2 peptides from apolipoprotein CI (APOCI), which was found to be in higher abundance in patient sera as compared to controls, and **(B)** MS/MS spectra of 2 peptides from elastase, neutrophil expressed (ELANE), which was downregulated in patient sera as compared to controls. (Insets) Relative intensity of reporter ions (m/z; control serum samples 114, chikungunya serum samples 115) from MS/MS fragmentation.

**Figure 3:**
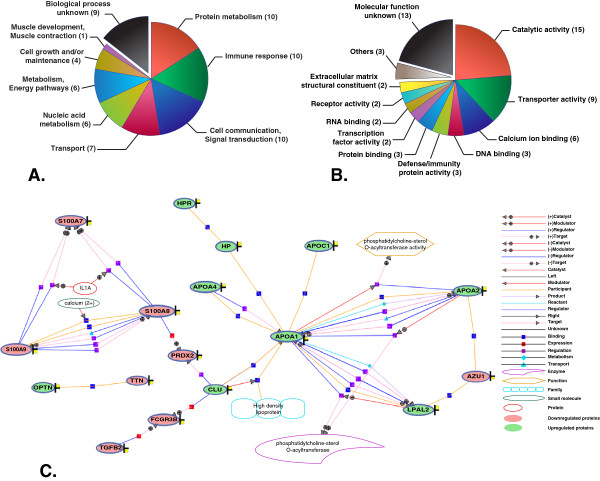
**Functional analysis. (A)** molecular function; and **(B)** biological process of differentially expressed proteins based on gene ontology. **(C)** Biological network of differentially regulated proteins.

We found 63 proteins to be differentially expressed (≥ 2-fold) (Figure 1) between the serum samples from patients and controls. Of these, 28 differentially expressed proteins were identified by multiple peptides and 17 protein identifications were based on single peptide evidence with multiple PSMs. Thirty five of these proteins were more abundant in chikungunya patient sera when compared to that from controls, while the remaining 28 proteins were less abundant (Table 1 and Table 2; Figure 2). We retrieved information on molecular functions and biological processes for the identified proteins from Human Protein Reference Database (HPRD) (http://www.hprd.org) [32,33]. The differentially expressed proteins identified in this study were found to be involved in biological processes such as protein metabolism, immune response, cell communication and transport and included molecules with catalytic and transporter activities (Figure 3A and 3B). We also subjected these differentially expressed proteins into a network analysis using GeneSpring software (version 12.5), which revealed an interaction network of 17 differentially expressed proteins, including apolipoproteins and S100A family proteins (Figure 3C). Functional analysis of these differentially expressed proteins revealed the overexpression of RNA binding proteins such as ATP-dependent RNA helicase (DDX23), sterile alpha motif domain-containing protein 3 (SAMD3); and extracellular matrix structural constituent proteins such as cartilage oligomeric matrix protein (COMP) and chitinase-3-like protein 1 (CHI3L1). In contrast, DNA binding proteins such as histones (HIST2H3C and HIST1H4I) and tet methylcytosine dioxygenase 1 (TET1), along with macrophage receptor (MARCO) and low affinity immunoglobulin gamma Fc region receptor III-B (FCGR3B); and serine-type peptidases such as elastase, neutrophil expressed (ELANE), myeloblastin precursor (PRTN3) and cathepsin preprotein (CTSG) were found to be downregulated.

**Table 1:** Partial list of proteins overexpressed in serum samples of chikungunya patients

	**Protein**	**Gene symbol**	**RefSeq accession**	**Description**	**Fold change (chikungunya/control)**
1	SH3 and PX domain-containing protein 2B	*DLST*	NP_001017995.1	It is an adapter protein and is involved in cell adhesion and migration of numerous cell types. It interacts with Fas-ligand, which is a cytotoxic effector molecule of T and NK cells.	**6**
2	Optineurin	*OPTN*	NP_001008214.1	It is a coiled-coil containing protein that interacts with viral protein and may utilize tumor necrosis factor-alpha or Fas-ligand pathways to mediate apoptosis, inflammation or vasoconstriction.	**5**
3	Spermine synthase	*SMS*	NP_004586.2	Its deficiency is shown to impair neurodevelopment and cognitive ability.	**5**
4	Succinyl-CoA ligase [ADP-forming] subunit beta, mitochondrial	*SUCLA2*	NP_003841.1	It is a mitochondrial matrix enzyme that hydrolyzes ATP to convert succinate to succinyl-CoA.	**4**
5	Apolipoprotein A-IV	*APOA4*	NP_000473.2	Apolipoproteinis an activator of lecithin-cholesterol acyltransferase.	**4**
6	Apolipoprotein C-I	*APOC1*	NP_001636.1	It is known to be activated upon differentiation of monocytes to macrophages.	**4**
7	Probable ATP-dependent RNA helicase DDX23	*DDX23*	NP_004809.2	It is a component of the U5 snRNP complex and has been reported to be upregulated upon induction of viral replication.	**3**
8	Haptoglobin isoform 1 preproprotein	*HP*	NP_005134.1	Haptoglobin binds to free plasma hemoglobin, and enables hemoglobin degradation. It also prevents loss of iron through kidneys and protects damage of kidneys from hemoglobin.	**3**
9	Glutamyl aminopeptidase	*ENPEP*	NP_001968.3	It is an aminopeptidase and is involved in the regulation of blood pressure.	**2**
10	Clusterin	*CLU*	NP_001822.3	It is a secreted chaperone. It is known to be involved in events such as cell death and neurodegenerative disorders.	**2**

**Table 2:** Partial list of proteins downregulated in serum samples of chikungunya infected patients

	**Protein**	**Gene symbol**	**RefSeq accession**	**Description**	**Fold change (control/ chikungunya)**
1	Azurocidin preproprotein	*AZU1*	NP_001691.1	It is an azurophil granule antibiotic protein, with monocyte chemotactic and antibacterial activity.	**2**
2	Peroxiredoxin-2 isoform a	*PRDX2*	NP_005800.3	It plays an antioxidant protective role in cells, and it may contribute to the antiviral activity of CD8 (+) T-cells.	**2**
3	Annexin A1	*ANXA1*	NP_000691.1	It is a calcium-dependent phospholipid binding proteins with potential anti-inflammatory activity.	**3**
4	Lipocalin-1 isoform 1	*LCN1*	NP_002288.1	Lipocalins are extracellular transport proteins that bind to a variety of hydrophobic ligands. Lipocalins are overproduced in response to multiple stimuli including infection and stress.	**4**
5	Protein S100-A9	*S100A9*	NP_002956.1	It is a calcium-binding protein and a member of the S100 family, involved in the regulation of cell cycle progression and differentiation.	**4**
6	Neutrophil elastase preproprotein	*ELANE*	NP_001963.1	It is a serine protease, which hydrolyzes proteins within azurophil granules and that of extracellular matrix following the protein’s release from activated neutrophils. The enzyme may play a role in degenerative and inflammatory diseases.	**5**
7	Myeloblastin	*PRTN3*	NP_002768.3	It is a serine protease found in the azurophilic granules in neutrophil granulocytes.	**6**
8	Cathepsin G preproprotein	*CTSG*	NP_001902.1	It is a member of the peptidase S1 protein family and is found in azurophil granules of neutrophilic polymorphonuclear leukocytes. It may participate in the killing and digestion of engulfed pathogens, and in connective tissue remodeling at sites of inflammation.	**6**
9	Protein S100-A7	*S100A7*	NP_002954.2	It is a member of S100 family of proteins but lacks calcium binding ability. It is markedly over-expressed in the skin lesions of psoriatic patients, but is excluded as a candidate gene for familial psoriasis susceptibility.	**7**
10	Phosphatidylinositide phosphatase SAC1	*SACM1L*	NP_054735.3	Sac1 is a phosphoinositide lipid phosphatase that removes the phosphate residue from the inositol head group of PI(4)P.	**16**

Serum is a challenging body fluid for mass spectrometry-based proteomic investigations. To our knowledge, there is no published report of an unbiased global investigation of serum proteome in the context of chikungunya fever apart from limited targeted quantitative studies to measure the circulating levels of certain cytokines, chemokines and growth factors (e.g. IL-6, IL-8, IL-beta) [22-25]. Our study presents the first account of the changes in serum protein levels in chikungunya infected individuals. Using 2D gel electrophoresis and mass spectrometry, 6 proteins including apolipoprotein A-IV (APOA4) and serotransferrin (TF) had been reported to be upregulated from tissue lysates of brain and liver of mice infected with chikungunya virus [20]. We also observed the overexpression of APOA4 in serum of chikungunya infected patients. Although we identified serotransferrin from serum, we did not find it to be differentially expressed. Of the three proteins reported to be downregulated in the mouse study, expression of catalase was in agreement with our study.

**Figure 4:**
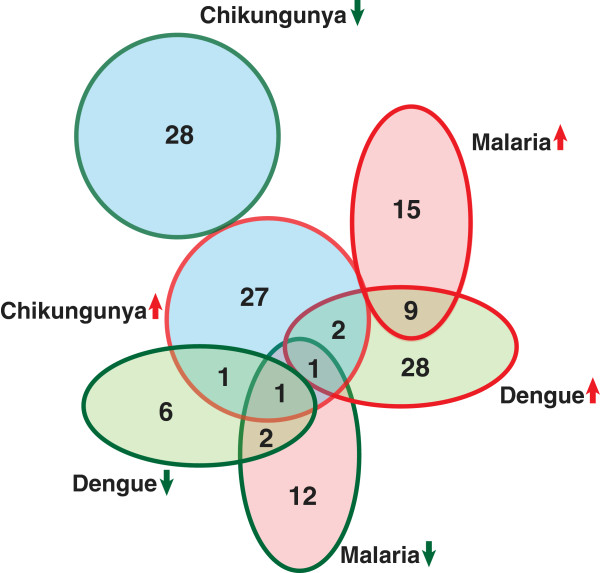
Overlap of proteins differentially expressed in serum samples of chikungunya patients, with that of differentially expressed proteins reported in dengue and malaria patients.

Proteins such as apolipoprotein A1 (APOA1), haptoglobin related protein (HPR) and clusterin (CLU) were found to be overexpressed in chikungunya serum samples. These three proteins were also reported to be overexpressed in serum samples of dengue infected individuals when compared to that of controls (Figure 4) [34,35]. However, clusterin has previously been reported to be downregulated in serum of malaria patients (Figure 4) [36,37]. Apolipoprotein C1 (APOC1) and APOA4 were found to be overexpressed in our study while they have been reported to be downregulated in serum of dengue infected individuals (Figure 4) [34,35]. APOA4 has previously been shown to be downregulated in malaria patients (Figure 4) [36,37].

We observed the downregulation of proteins that are known to be involved in the chemotaxis of neutrophils and phagocytes as a part of immune response to infection, including azurocidin (AZU1), annexin A1 (ANXA1), CTSG, S-100 calcium binding proteins S100A7, S100A8, S100A9 and transforming growth factor, beta 2 (TGFB2) from chikungunya serum samples. Proteins involved in clathrin mediated endocytosis such as APOC1, APOA4, APOA1, APOA2 and clusterin were abundantly expressed in patient serum samples. Optineurin (OPTN), which has been reported to be highly expressed in ocular tissues, was also overexpressed in the serum samples in the context of chikungunya when compared to that of controls. Optineurin has also been reported to have anti-viral signaling role brought about by the negative regulation of interferon beta [38]. It may be associated with the ocular manifestations reported in chikungunya [39,40]. Haptoglobin is another acute phase protein that was overexpressed in patient serum samples [41].

Arthralgia occurs in the majority (~90%) of chikungunya patients. Prolonged inflammatory responses have been reported to be the cause of arthritogenic symptoms in chikungunya [23,25]. Gene expression profiling studies in mouse models of chikungunya have demonstrated significant overlap of differentially expressed genes in chikungunya and rheumatoid arthritis [21]. Of the proteins identified in this study, several differentially expressed proteins are known to be associated with arthritis including ANXA1, APOA1, chitinase 3 like-1 (CHI3L1), CLU, COMP, HP, ICOS ligand (ICOSLG) and lactotransferrin (LTF), among others [42-45].

Apolipoproteins are known to be involved in the lipid metabolism, particularly in the esterification of cholesterol. An increase in the serum levels of apolipoproteins (APOA1, APOA2, APOA4 and APOC1) was observed in chikungunya patients, which suggests that alterations in lipid metabolism occur in the context of chikungunya. Although the exact functions of APOA4 remain unknown, serum levels of APOA4 are known to be associated with the higher consumption of dietary lipids [46]. Lipoprotein metabolism has been shown to be modulated in case of viral infections including hepatitis C virus. Apolipoproteins have been shown to mediate viral entry into hepatocytes through the formation of lipo-viro particles and these viral particles are shown to circulate in plasma [47-50].

Rashes are a common symptom of chikungunya infection. Maculopapular rashes have been reported in ~50% of the cases. There have been reports of other skin manifestations such as pigmentation and dermatitis in chikungunya patients [39]. In this context, it is interesting to note that we identified several proteins associated with oncostatin M (OSM) pathway as differentially expressed in chikungunya, as this pathway has been reported to have a unique role in hypersensitivity, dermatitis and psoriasis [51,52]. Although OSM was itself not identified in our study, proteins including HP, CHI3L1, selenium binding protein 1 (SELENBP1), S-100 calcium binding proteins (S100A7, S100A8 and S100A9), and ANXA1, which were known to be regulated by OSM pathway, were identified with significant alterations in their expression level from this investigation [51,53-57].

In recent times, association of chikungunya with neurological manifestations such as encephalitis and meningoencephalitis has been reported, primarily in neonates. It has also been linked to mortality in chikungunya infected individuals [58-60]. Post-chikungunya, a few patients have also presented with Guillain-Barré syndrome (GBS), an acute inflammatory disorder affecting the peripheral nervous system [61]. Increased levels of APOA4 and HP observed in our study have been previously reported from the cerebrospinal fluid of patients with GBS and Huntington’s disease [62,63].

In isolated reports of neurological manifestations associated with chikungunya, documented from Kerala and Maharashtra in India, significant hyponatremia has been observed [64,65]. Hyponatremia has also been reported to be induced in infections such as malaria, dengue, HIV infection and hepatitis [66-70]. Although the exact mechanisms of the induction of hyponatremia in these conditions are not well understood, it is associated with hyperproteinemia and hyperlipidemia [68]. In this context, we observed overexpression of electrogenic sodium bicarbonate cotransporter 1 isoform 2 (SLC4A4) in serum samples of chikungunya patients, which is known to regulate the sodium influx along with sodium bicarbonate levels, thereby maintaining the pH of blood [71].

### Public availability of proteomic data

We have submitted the proteome data to Human Proteinpedia [31]. The data can be accessed using the following URL: http://www.humanproteinpedia.org/data_display?exp_id=00708.

We have also submitted the proteome data to the ProteomeXchange Consortium (http://proteomecentral.proteomexchange.org) via the PRIDE partner repository [30] with the dataset identifier PXD000234.

## Conclusions

This is the first report of an unbiased quantitative proteomic profiling of serum from chikungunya patients. It provides an initial account of changes in serum proteins, which can initiate new avenues in chikungunya research to further the understanding of the clinical manifestations. Our study has identified a number of differentially expressed proteins, which can be chosen for further validation. In particular, these proteins could be assessed further for their suitability as candidate biomarkers to distinguish various vector-borne diseases and for measuring the severity of the disease progression.

## Methods

### Patient and sample details

Patients with 1–10 days of illness and typical symptoms like polyarthralgia and fever were worked up for chikungunya infection. Blood samples from patients and age and sex matched controls were obtained after informed consent and approval of the institutional ethics review panel at the All India Institute of Medical Sciences, New Delhi, India (Table 3). Serum was isolated from the blood sample using standard centrifugation procedures. The samples were confirmed positive for chikungunya infection and negative for dengue infection using anti-CHIKV-IgM capture and anti-Dengue-IgM capture ELISA kits, respectively (supplied by National Institute of Virology, Pune, India). Further confirmation of infection was performed by amplification of chikungunya virus specific E1 glycoprotein transcripts using RT-PCR [72]. Four samples that tested negative were used as controls.

**Table 3:** Details of the samples used in this study, with the clinical presentations at the time of collection

	**Sample ID**	**Age**	**Sex**	**Days of illness (chikungunya)**	**Fever**	**Arthralgia**	**Rashes**
**Control samples**							
**1**	4915	20	Female	N/A	No	No	No
**2**	5308	20	Male	N/A	No	No	No
**3**	4917	40	Female	N/A	No	No	No
**4**	5444	32	Female	N/A	No	No	No
**Chikungunya infected patient samples (IgM and PCR positive)**							
**1**	V-10 4706	63	Male	7	Positive	Positive	No
**2**	V-10 4669	53	Female	7	Positive	Positive	No
**3**	V-10 4663	28	Female	6	Positive	Positive	No
**4**	V-10 4696	45	Female	5	Positive	Positive	No
**5**	V-10 4629	45	Male	7	Positive	No	No

### RNA extraction and reverse transcriptase PCR (RT-PCR)

RNA from serum samples was isolated using Qiagen Viral RNA extraction kit and stored at -70°C until use. cDNA synthesis was carried out using avian myeloblastosis virus reverse transcriptase and PCR was carried out using published primers to generate a 294 bp fragment from the E1 gene of CHIKV [72]. The PCR products were visualized in 2% agarose gel using ethidium bromide under Gel Doc™ XR + System (Biorad) (Additional file 1: Figure S1). The identity of the amplicons was further confirmed by sequencing.

### Protein isolation and depletion of abundant proteins

Total protein estimation of these serum samples was performed using BCA assay (Pierce®. Cat#: 23225) and normalization of protein amounts were confirmed by SDS-PAGE. Equivalent amounts of protein from each of the infected and control serum samples were pooled separately. Comparable amounts of pooled serum and control samples were further depleted of the high abundant proteins of serum, using a Multiple Affinity Removal Spin Cartridge for human serum (Agilent Technologies, Santa Clara, CA. Cat#: 5188–5230) as per manufacturer’s instructions.

### Peptide labeling and fractionation

After depletion, equal amounts of protein from each sample were processed for further analysis as described earlier [73]. Briefly, about 70 μg of proteins from each set was reduced using 2 μL of tris-(2-carboxyethyl)phosphine at 60°C for 1 hour, followed by alkylation of cysteine residues using methyl methanethiosulfonate for 10 minutes at room temperature. The samples were then digested using sequencing grade trypsin (1:20) (Promega, Madison, WI. Cat#: V5111) at 37°C for 12 hours. The digestion mixture volume was reduced by vacuum drying to about 40 μL and the peptides in each tube were labeled with iTRAQ labeling reagents (Applied Biosystems, Cat#: 4352135) following the manufacturer’s protocol. The infected and control serum samples were labeled with iTRAQ labels, which will lead to 115 and 114 reporter ions upon ionization, respectively. After incubating the samples with the labels for an hour, the reaction was quenched by addition of 150 μL milliQ water and vacuum dried. The labeled peptides from infected and control serum samples were reconstituted in 5 mM potassium phosphate buffer, 25% acetonitrile (pH 2.7) (solvent A), pooled and subjected to strong cation exchange (SCX) chromatography as described earlier [74]. The labeled peptides were fractionated using polysulfoethyl A column (PolyLC, Columbia, MD. Cat#: 204SE0502) (200 Ǻ, 5 μm, 200 × 4.6 mm) on an Agilent 1200 infinity series HPLC system consisting of a binary pump, external sample injector, UV detector and a fraction collector. Fractionation was carried out for a period of 50 min using a gradient of increasing salt concentration of up to 350 mM KCl in solvent A. The eluate was pooled to obtain 22 fractions, dried in vacuum dryer, reconstituted in 40 μL of 0.1% trifluoroacetic acid and desalted using C18 (3 M Empore high-performance extraction disks) stage-tips.

### LC-MS/MS and data analysis

The fractions were analyzed on LTQ-Orbitrap Velos ETD mass spectrometer (Thermo Scientific, Bremen, Germany) interfaced with Easy-nLCII (Thermo Scientific, Bremen, Germany). Peptides were initially enriched on a reversed phase liquid chromatography (RPLC) pre-column (2 cm, 5 μ – 100 Ǻ), followed by separation on an analytical column (11 cm, 3 μ – 100 Ǻ) made with magic AQ C18 material (Michrom Bioresources, Inc, Auburn, CA) packed in-house. The peptides were sprayed using nano electro spray emitter tip of 10 μ (New Objective, Woburn, MA). The solvent system used includes 0.1% aqueous formic acid as solvent A and 100% acetonitrile, 0.1% formic acid as solvent B. The peptides were loaded on the trap column using 97% solvent A, followed by resolution on the analytical column using a linear gradient of 5-30% solvent B for 70 min at a constant flow rate of 0.35 μL/min. The spray voltage and heated capillary temperature were set to 2.0 kV and 220°C, respectively and data was acquired in a data dependent manner. Fifteen most intense precursor ions were selected for fragmentation from each MS scan. MS and MS/MS scans were acquired in an Orbitrap mass analyzer at a resolution of 60,000 at 400 m/z and 15,000, respectively. The peptides were fragmented by higher energy collision dissociation with normalized collision energy of 41%. The automatic gain control (AGC) for full FT MS was set to 1 million ions and for FT MS/MS was set to 0.1 million ions with maximum accumulation time of 200 ms and 500 ms, respectively.

The data obtained was searched against the human RefSeq 52 proteins using Proteome Discoverer, version 1.3.0.339 (Thermo Fischer Scientific, Bremen, Germany) workflow. The workflow consisted of spectrum selector and reporter ion quantification nodes in addition to SEQUEST and Mascot search nodes. Similar parameters were used in all the searches with trypsin as enzyme allowing a single missed cleavage. Other parameters include methylthiol modification of cysteine, iTRAQ labels at the peptide N-terminus and Lysine residues as static modifications and oxidation of methionine as variable modification. A mass tolerance of 20 ppm and 0.1 Da were used for the precursor ion and fragment ions, respectively with a signal to noise ratio of 1.5 for a precursor mass range of 350–10000 Da. A false discovery rate (FDR) of 1% was applied to the results. The differentially expressed proteins obtained from these searches were analyzed further for functional categorization based on biological processes and molecular function using annotations in HPRD [32]. The differentially expressed proteins were further analyzed using the GeneSpring analysis software version 12.5 (Agilent Biosystems, Santa Clara, CA), as described previously [75,76]. We also integrated the interaction data from NetPath (http://netpath.org) and HPRD in the analysis using GeneSpring software [77]. Literature survey was performed to identify serum based proteome studies in dengue, chikungunya and malaria. The differentially expressed proteins obtained in this study were then compared with the differentially expressed proteins reported in dengue and malaria by other groups [34-37].

## Abbreviations

WHO: World Health Organization; CDC: Centre for Disease Control; iTRAQ: isobaric tag for Relative and Absolute Quantitation; PSM: Peptide Spectral Match; PPD: Plasma Proteome Database; HPRD: Human Protein Reference Database; GBS: Guillain-Barré syndrome; FDR: False Discovery Rate.

## Competing interests

The authors declare that they have no competing interests.

## Authors’ contributions

HCH, AP, SB, JN and TSKP conceived the idea and planned the study. SB enrolled serum samples for this study. AG, PKS and SB performed diagnostic assays and confirmed the diagnosis. SKS and VNP processed the samples for mass spectrometry and VNP performed mass spectrometry. SKS and VNP analyzed mass spectrometry derived data. SKS prepared figures and tables. SKS, HCH, AP and TSKP wrote the manuscript. JN and AP provided critical inputs and revised the manuscript. All authors read and approved the final manuscript.

## Supplementary Material

Additional file 1: Figure S1 Confirmation of chikungunya infection by RT-PCR. Chikungunya infection was confirmed by the presence of a band corresponding to 294 bp after PCR amplification. Click here for file
