# Supplementary figures and images for: Proteomic profiling of serum samples from chikungunya-infected patients provides insights into host response
Source: Clin Proteomics. 2013 Oct 14;10(1):14. doi: 10.1186/1559-0275-10-14 (PMC3879382; doi:10.1186/1559-0275-10-14)

**Additional Figure 1**

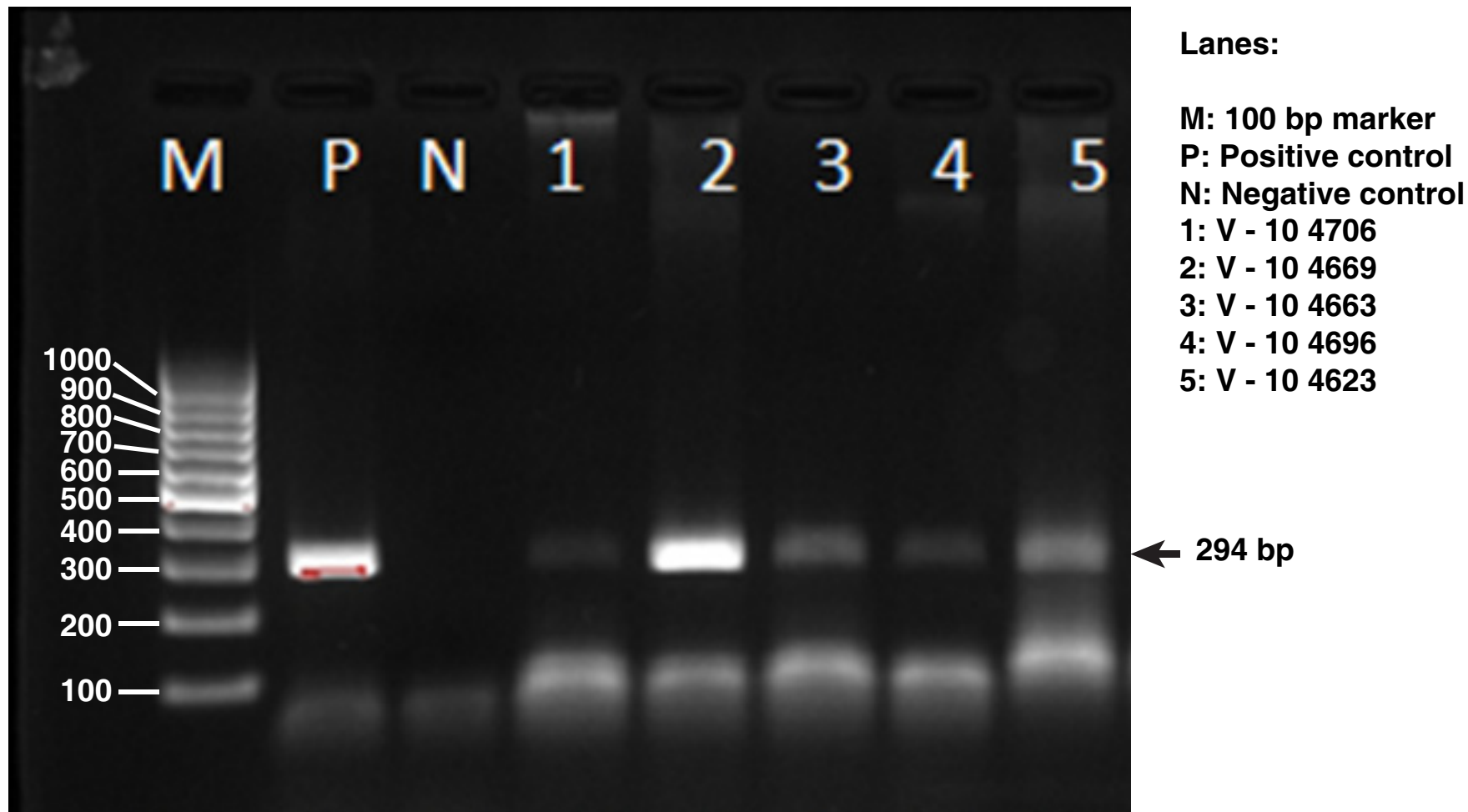

Supplement: Additional file 1: Figure S1 — Confirmation of chikungunya infection by RT-PCR. Chikungunya infection was confirmed by the presence of a band corresponding to 294 bp after PCR amplification. [file 1559-0275-10-14-S1.pdf]
